# Bis[(*E*)-3-(4-meth­oxy­phen­yl)prop-2-enoato]triphenyl­anti­mony(V) benzene monosolvate

**DOI:** 10.1107/S1600536813004674

**Published:** 2013-02-23

**Authors:** Pavel V. Andreev, Nikolay V. Somov, Olga S. Kalistratova, Alexey V. Gushchin, Evgeny V. Chuprunov

**Affiliations:** a Department of Physics, N. I. Lobachevsky State University of Nizhni Novgorod, 603950, pr. Gagarina 23-3, Nizhni Novgorod, Russian Federation; b Department of Chemistry, N. I. Lobachevsky State University of Nizhni Novgorod, 603950, pr. Gagarina 23-2, Nizhni Novgorod, Russian Federation

## Abstract

The asymmetric unit of the title compound, [Sb(C_6_H_5_)_3_(C_10_H_9_O_3_)_2_]·C_6_H_6_, contains one organometallic mol­ecule and one benzene mol­ecule that is disordered over two sets of sites with an occupancy ratio of 0.556 (15):0.444 (15). The Sb^V^ atom is in a distorted trigonal–bipyramidal environment with the carboxyl­ate O atoms in axial positions and phenyl C atoms in the equatorial plane. As a result of additional Sb⋯O inter­actions, one of the C—Sb—C angles is widened to 140.19 (6)°.

## Related literature

For the chemistry of triphenyanti­mony diacyl­ates, see: Gushchin *et al.* (2011 ▶). For thermodynamic properties of tri­phenyl­anti­mony diacyl­ates, see: Letyanina *et al.* (2012 ▶); Markin *et al.* (2011 ▶) and for their applications, see: Dodonov *et al.* (2004 ▶). For a closely related structure, see: Belsky (1996 ▶).
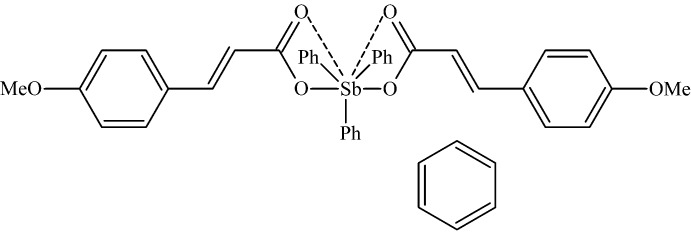


## Experimental

### 

#### Crystal data

[Sb(C_6_H_5_)_3_(C_10_H_9_O_3_)_2_]·C_6_H_6_
*M* *_r_* = 785.5
Triclinic, 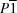

*a* = 11.2047 (1) Å
*b* = 11.2337 (1) Å
*c* = 15.0317 (2) Å
α = 91.125 (1)°
β = 94.936 (1)°
γ = 95.496 (1)°
*V* = 1875.62 (3) Å^3^
*Z* = 2
Mo *K*α radiation
μ = 0.78 mm^−1^
*T* = 293 K
0.22 × 0.16 × 0.07 mm

#### Data collection

Agilent Xcalibur Sapphire3 CCD diffractometer
Absorption correction: multi-scan (*CrysAlis RED*; Agilent, 2011 ▶) *T*_min_ = 0.841, *T*_max_ = 1.000
28524 measured reflections
7595 independent reflections
7254 reflections with *I* > 2σ(*I*)
*R*_int_ = 0.022

#### Refinement

*R*[*F* ^2^ > 2σ(*F* ^2^)] = 0.020
*wR*(*F* ^2^) = 0.052
*S* = 1.09
7595 reflections
551 parameters
78 restraints
H-atom parameters constrained
Δρ_max_ = 0.34 e Å^−3^
Δρ_min_ = −0.25 e Å^−3^

### 

Data collection: *CrysAlis CCD* (Agilent, 2011 ▶); cell refinement: *CrysAlis CCD*; data reduction: *CrysAlis RED* (Agilent, 2011 ▶); program(s) used to solve structure: *SHELXS97* (Sheldrick, 2008 ▶); program(s) used to refine structure: *SHELXL97* (Sheldrick, 2008 ▶); molecular graphics: *Mercury* (Macrae *et al.*, 2006 ▶); software used to prepare material for publication: *publCIF* (Westrip, 2010 ▶).

## Supplementary Material

Crystal structure: contains datablock(s) I, global. DOI: 10.1107/S1600536813004674/yk2083sup1.cif

Structure factors: contains datablock(s) I. DOI: 10.1107/S1600536813004674/yk2083Isup2.hkl

Additional supplementary materials:  crystallographic information; 3D view; checkCIF report

## Figures and Tables

**Fig. 1.**
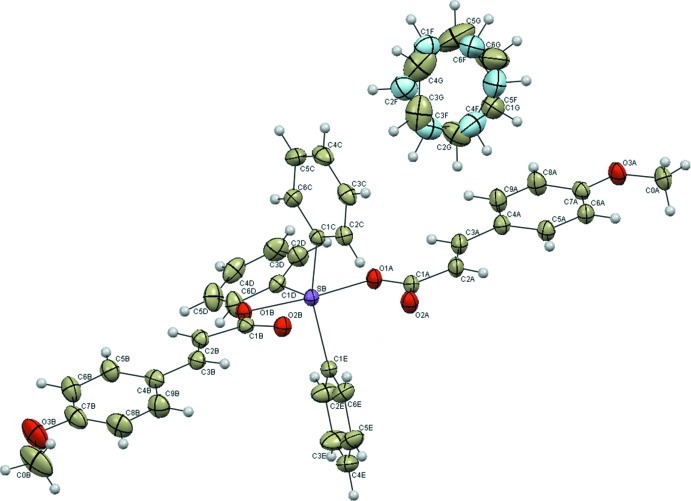
The structure of an asymmetric part of the title compound with the atom numbering scheme. Displacement ellipsoids are drawn at the 30% probability level. H atoms are presented as small spheres of arbitrary radius. Two orientations of the solvent benzene molecule are shown by different colours.

## supplementary crystallographic information

### Comment

The triphenylantimony bis(*p*-methoxycinnamate), C_44_H_39_O_6_Sb, belongs to the family of triphenylantimony diacylates. It contains two double bonds C=C in its molecule, due to which it can be used for polymerization filling of polystyrene and polymethylmethacrylate. Especially, this compound is a very promising monomer for developing metal-containing organic scintillators, which have recently attracted much attention in high-energy physics. It was found that the participation of both acrylate groups in polymerization leads to spatial cross-linking, considerably decreasing the thermooxidative destruction of the resulting polymer (Dodonov *et al.*, 2004). Organic glasses based on triphenylantimony diacrylate and methylmethacrylate having increased fungal resistance are now available (Dodonov *et al.*, 2004).

In the organometallic molecule of the title compound, the O–Sb–O angle is 172.81 (4)°, and the Ph–Sb–Ph angles are 107.48 (6)°, 112.33 (6)°, and 140.19 (6)°. Such difference from 120° is typical of triphenylantomony diacylates because of additional Sb···O contacts. For example, similar geometry was observed in triphenylantimony dimethacrylate (Gushchin *et al.*, 2011). The Sb–O2A and Sb–O2B distances being 2.937 (13) Å and 2.900 (13) Å, respectively, are significantly shorter than the sum of the van der Waals radii of these atoms (3.60 Å). The solvent benzene molecule was modelled as disordered over two sets of sites with an occupancy ratio of 0.556 (15):0.444 (15).

### Experimental

The synthesis was carried out on the oxidation addition reaction of triphenylantimony, *p*-methoxycinnamic acid and hydrogen peroxide. To a solution of 0.7 ml of 31.2% aqueous hydrogen peroxide and 2.46 g of *p*-methoxycinnamic acid in 35 ml of tetrahydrofuran was added a solution of 2.2 g of triphenylantimony. The mixture was kept for 12 h at room temperature. The colourless crystals formed were filtered off and dried to obtain 1.63 g (95%) of triphenylantimony di-*p*-methoxycinnamate. The product was recrystallized twice from chloroform-hexane mixture (1:4), m.p. 178°C. Crystal for X-ray diffraction analysis was obtained from benzene solution.

### Refinement

H atoms were positioned geometrically (C—H = 0.95–1.00 Å) and refined using a riding model with the *U*_iso_(H) = 1.2Ueq(C) (1.5Ueq(C) for methyl groups).

### Crystal data

**Table:**

[Sb(C_6_H_5_)_3_(C_10_H_9_O_3_)_2_]·C_6_H_6_	*Z* = 2
*M**_r_* = 785.5	*F*(000) = 804
Triclinic, *P*1	*D*_x_ = 1.391 Mg m^−^^3^
Hall symbol: -P 1	Mo *K*α radiation, λ = 0.71073 Å
*a* = 11.2047 (1) Å	Cell parameters from 20589 reflections
*b* = 11.2337 (1) Å	θ = 3.6–33.0°
*c* = 15.0317 (2) Å	µ = 0.78 mm^−^^1^
α = 91.125 (1)°	*T* = 293 K
β = 94.936 (1)°	Bulk, colourless
γ = 95.496 (1)°	0.22 × 0.16 × 0.07 mm
*V* = 1875.62 (3) Å^3^	

### Data collection

**Table:**

Agilent Xcalibur Sapphire3 CCD diffractometer	7595 independent reflections
Graphite monochromator	7254 reflections with *I* > 2σ(*I*)
Detector resolution: 16.0302 pixels mm^-1^	*R*_int_ = 0.022
ω scans	θ_max_ = 26.4°, θ_min_ = 3.6°
Absorption correction: multi-scan (*CrysAlis RED*; Agilent, 2011)	*h* = −13→13
*T*_min_ = 0.841, *T*_max_ = 1.000	*k* = −14→14
28524 measured reflections	*l* = −18→18

### Refinement

**Table:**

Refinement on *F*^2^	Primary atom site location: structure-invariant direct methods
Least-squares matrix: full	Secondary atom site location: difference Fourier map
*R*[*F*^2^ > 2σ(*F*^2^)] = 0.020	Hydrogen site location: inferred from neighbouring sites
*wR*(*F*^2^) = 0.052	H-atom parameters constrained
*S* = 1.09	*w* = 1/[σ^2^(*F*_o_^2^) + (0.0248*P*)^2^ + 0.4754*P*] where *P* = (*F*_o_^2^ + 2*F*_c_^2^)/3
7595 reflections	(Δ/σ)_max_ = 0.001
551 parameters	Δρ_max_ = 0.34 e Å^−^^3^
78 restraints	Δρ_min_ = −0.25 e Å^−^^3^

### Special details

**Table:**

Geometry. All s.u.'s (except the s.u. in the dihedral angle between two l.s. planes) are estimated using the full covariance matrix. The cell s.u.'s are taken into account individually in the estimation of s.u.'s in distances, angles and torsion angles; correlations between s.u.'s in cell parameters are only used when they are defined by crystal symmetry. An approximate (isotropic) treatment of cell s.u.'s is used for estimating s.u.'s involving l.s. planes.
Refinement. Refinement of *F*^2^ against ALL reflections. The weighted *R*-factor *wR* and goodness of fit *S* are based on *F*^2^, conventional *R*-factors *R* are based on *F*, with *F* set to zero for negative *F*^2^. The threshold expression of *F*^2^ > σ(*F*^2^) is used only for calculating *R*-factors(gt) *etc*. and is not relevant to the choice of reflections for refinement. *R*-factors based on *F*^2^ are statistically about twice as large as those based on *F*, and *R*- factors based on ALL data will be even larger.

### Fractional atomic coordinates and isotropic or equivalent isotropic displacement parameters (Å^2^)

**Table:**

	*x*	*y*	*z*	*U*_iso_*/*U*_eq_	Occ. (<1)
Sb	0.218385 (8)	0.298563 (8)	0.372245 (6)	0.03567 (4)	
C1C	0.21079 (14)	0.48201 (14)	0.39945 (9)	0.0382 (3)	
C2C	0.31247 (16)	0.56356 (15)	0.40952 (11)	0.0460 (4)	
H2C	0.3885	0.5381	0.406	0.055*	
C3C	0.2993 (2)	0.68249 (16)	0.42481 (12)	0.0572 (5)	
H3C	0.3667	0.7381	0.4304	0.069*	
C4C	0.1873 (2)	0.71945 (17)	0.43181 (14)	0.0650 (5)	
H4C	0.1795	0.8	0.4426	0.078*	
C5C	0.0867 (2)	0.63916 (19)	0.42315 (15)	0.0643 (5)	
H5C	0.0112	0.665	0.4286	0.077*	
C6C	0.09797 (16)	0.51918 (17)	0.40616 (12)	0.0503 (4)	
H6C	0.0301	0.4642	0.3993	0.06*	
C1D	0.04561 (14)	0.20684 (15)	0.33960 (11)	0.0425 (3)	
C2D	−0.02135 (17)	0.22894 (19)	0.26182 (14)	0.0592 (5)	
H2D	0.0074	0.2874	0.2239	0.071*	
C3D	−0.13217 (19)	0.1638 (2)	0.23982 (16)	0.0729 (6)	
H3D	−0.1776	0.1797	0.1874	0.087*	
C4D	−0.17495 (18)	0.0770 (2)	0.29407 (16)	0.0706 (6)	
H4D	−0.2484	0.0327	0.2782	0.085*	
C5D	−0.1102 (2)	0.0556 (2)	0.37109 (17)	0.0767 (7)	
H5D	−0.1398	−0.003	0.4086	0.092*	
C6D	0.00029 (19)	0.1203 (2)	0.39475 (14)	0.0643 (5)	
H6D	0.0439	0.1051	0.4481	0.077*	
C1E	0.34500 (14)	0.17259 (14)	0.36646 (10)	0.0410 (3)	
C2E	0.3032 (2)	0.06290 (19)	0.32898 (17)	0.0712 (6)	
H2E	0.2231	0.0481	0.3064	0.085*	
C3E	0.3800 (2)	−0.0262 (2)	0.3246 (2)	0.0848 (8)	
H3E	0.3518	−0.1004	0.2984	0.102*	
C4E	0.4962 (2)	−0.0051 (2)	0.35859 (17)	0.0730 (6)	
H4E	0.5474	−0.0653	0.3564	0.088*	
C5E	0.5381 (2)	0.1036 (2)	0.39573 (15)	0.0700 (6)	
H5E	0.6182	0.1174	0.4186	0.084*	
C6E	0.46365 (17)	0.19373 (19)	0.39993 (13)	0.0573 (4)	
H6E	0.4931	0.2682	0.4251	0.069*	
C0A	0.2877 (2)	0.5977 (2)	−0.33971 (13)	0.0671 (5)	
H0A1	0.2631	0.6095	−0.4014	0.101*	
H0A2	0.3509	0.5456	−0.3357	0.101*	
H0A3	0.3164	0.6733	−0.311	0.101*	
C1A	0.32163 (15)	0.38933 (15)	0.21471 (10)	0.0445 (4)	
O1A	0.21965 (10)	0.33407 (11)	0.23530 (7)	0.0469 (3)	
C2A	0.32431 (16)	0.42905 (17)	0.12203 (11)	0.0507 (4)	
H2A	0.3973	0.4626	0.1043	0.061*	
O2A	0.40867 (11)	0.40838 (13)	0.26879 (8)	0.0563 (3)	
C3A	0.23030 (17)	0.42025 (17)	0.06284 (11)	0.0504 (4)	
H3A	0.1588	0.3848	0.0819	0.06*	
O3A	0.18849 (12)	0.54539 (14)	−0.29707 (8)	0.0616 (3)	
C4A	0.22464 (16)	0.45949 (16)	−0.02952 (11)	0.0474 (4)	
C5A	0.32322 (16)	0.50851 (17)	−0.06998 (11)	0.0507 (4)	
H5A	0.3972	0.521	−0.0364	0.061*	
C6A	0.31586 (16)	0.53964 (17)	−0.15873 (11)	0.0493 (4)	
H6A	0.3839	0.5723	−0.1842	0.059*	
C7A	0.20639 (16)	0.52176 (16)	−0.20900 (11)	0.0479 (4)	
C8A	0.10605 (18)	0.4750 (2)	−0.16940 (13)	0.0630 (5)	
H8A	0.0317	0.4642	−0.2027	0.076*	
C9A	0.11518 (18)	0.4445 (2)	−0.08156 (13)	0.0616 (5)	
H9A	0.0467	0.413	−0.0561	0.074*	
C0B	0.5056 (4)	0.1339 (3)	1.1230 (2)	0.1347 (15)	
H0B1	0.4902	0.1048	1.1809	0.202*	
H0B2	0.5418	0.215	1.1289	0.202*	
H0B3	0.5592	0.0849	1.0963	0.202*	
C1B	0.28748 (15)	0.29644 (14)	0.56128 (10)	0.0416 (3)	
O1B	0.19336 (10)	0.25803 (10)	0.50719 (7)	0.0445 (2)	
C2B	0.27826 (16)	0.26299 (15)	0.65452 (11)	0.0459 (4)	
H2B	0.2045	0.231	0.6719	0.055*	
O2B	0.37549 (11)	0.35276 (11)	0.53445 (8)	0.0503 (3)	
C3B	0.37252 (17)	0.27738 (15)	0.71443 (11)	0.0479 (4)	
H3B	0.4428	0.3156	0.6954	0.057*	
O3B	0.3990 (2)	0.1298 (2)	1.06980 (12)	0.1140 (7)	
C4B	0.37851 (18)	0.23995 (16)	0.80714 (11)	0.0500 (4)	
C5B	0.2787 (2)	0.19744 (19)	0.84934 (13)	0.0620 (5)	
H5B	0.203	0.1926	0.818	0.074*	
C6B	0.2886 (3)	0.1623 (2)	0.93644 (15)	0.0764 (6)	
H6B	0.22	0.1349	0.9635	0.092*	
C7B	0.3999 (3)	0.1674 (2)	0.98391 (14)	0.0741 (6)	
C8B	0.4988 (3)	0.2095 (2)	0.94436 (15)	0.0803 (7)	
H8B	0.5741	0.2139	0.9762	0.096*	
C9B	0.4887 (2)	0.2464 (2)	0.85619 (14)	0.0694 (6)	
H9B	0.5574	0.2759	0.8301	0.083*	
C1F	0.0632 (16)	0.9174 (17)	0.1729 (13)	0.088 (3)	0.556 (15)
H1F	0.0161	0.9705	0.1983	0.106*	0.556 (15)
C2F	0.0867 (12)	0.8159 (13)	0.2132 (10)	0.092 (3)	0.556 (15)
H2F	0.0597	0.7997	0.2689	0.111*	0.556 (15)
C3F	0.1514 (17)	0.7351 (12)	0.1718 (13)	0.093 (6)	0.556 (15)
H3F	0.1728	0.6673	0.2013	0.112*	0.556 (15)
C4F	0.1827 (13)	0.7544 (12)	0.0904 (13)	0.111 (5)	0.556 (15)
H4F	0.2173	0.6963	0.0592	0.134*	0.556 (15)
C5F	0.1634 (13)	0.8610 (14)	0.0527 (8)	0.159 (6)	0.556 (15)
H5F	0.1885	0.8781	−0.0034	0.19*	0.556 (15)
C6F	0.1086 (11)	0.9397 (10)	0.0966 (8)	0.125 (4)	0.556 (15)
H6F	0.1019	1.0145	0.0724	0.15*	0.556 (15)
C1G	0.1532 (9)	0.7766 (9)	0.0315 (8)	0.087 (3)	0.444 (15)
H1G	0.1787	0.7433	−0.0199	0.105*	0.444 (15)
C2G	0.173 (2)	0.7231 (14)	0.1135 (11)	0.095 (5)	0.444 (15)
H2G	0.2146	0.6553	0.1167	0.114*	0.444 (15)
C3G	0.132 (2)	0.769 (2)	0.1913 (13)	0.115 (11)	0.444 (15)
H3G	0.1408	0.729	0.2448	0.138*	0.444 (15)
C4G	0.0777 (19)	0.875 (2)	0.1863 (12)	0.131 (13)	0.444 (15)
H4G	0.0567	0.9114	0.2382	0.157*	0.444 (15)
C5G	0.0546 (16)	0.9287 (17)	0.1041 (12)	0.160 (8)	0.444 (15)
H5G	0.0125	0.9961	0.1007	0.192*	0.444 (15)
C6G	0.0950 (15)	0.8804 (10)	0.0272 (9)	0.135 (5)	0.444 (15)
H6G	0.0828	0.9177	−0.027	0.162*	0.444 (15)

### Atomic displacement parameters (Å^2^)

**Table:**

	*U*^11^	*U*^22^	*U*^33^	*U*^12^	*U*^13^	*U*^23^
Sb	0.03384 (6)	0.03966 (6)	0.03304 (6)	0.00126 (4)	0.00262 (4)	0.00237 (4)
C1C	0.0428 (8)	0.0399 (8)	0.0322 (7)	0.0039 (6)	0.0037 (6)	0.0038 (6)
C2C	0.0468 (9)	0.0481 (9)	0.0419 (8)	−0.0021 (7)	0.0039 (7)	0.0026 (7)
C3C	0.0738 (13)	0.0461 (9)	0.0485 (10)	−0.0071 (9)	0.0013 (9)	0.0022 (7)
C4C	0.0948 (16)	0.0439 (10)	0.0575 (11)	0.0122 (10)	0.0069 (11)	0.0019 (8)
C5C	0.0688 (13)	0.0622 (12)	0.0679 (13)	0.0273 (10)	0.0147 (10)	0.0078 (10)
C6C	0.0469 (9)	0.0517 (10)	0.0537 (10)	0.0080 (8)	0.0086 (7)	0.0064 (8)
C1D	0.0367 (8)	0.0447 (8)	0.0454 (8)	0.0006 (6)	0.0042 (6)	−0.0049 (7)
C2D	0.0446 (9)	0.0682 (12)	0.0621 (11)	−0.0004 (9)	−0.0050 (8)	0.0079 (9)
C3D	0.0460 (10)	0.0924 (16)	0.0756 (14)	0.0015 (11)	−0.0136 (10)	−0.0053 (12)
C4D	0.0431 (10)	0.0773 (14)	0.0873 (16)	−0.0135 (10)	0.0093 (10)	−0.0265 (12)
C5D	0.0687 (14)	0.0785 (15)	0.0768 (15)	−0.0302 (12)	0.0138 (11)	−0.0008 (12)
C6D	0.0619 (12)	0.0718 (13)	0.0539 (11)	−0.0190 (10)	0.0016 (9)	0.0058 (9)
C1E	0.0426 (8)	0.0410 (8)	0.0403 (8)	0.0059 (6)	0.0071 (6)	0.0040 (6)
C2E	0.0550 (11)	0.0527 (11)	0.1040 (18)	0.0040 (9)	0.0002 (11)	−0.0137 (11)
C3E	0.0812 (16)	0.0427 (11)	0.131 (2)	0.0073 (11)	0.0140 (15)	−0.0140 (12)
C4E	0.0738 (14)	0.0593 (12)	0.0937 (17)	0.0285 (11)	0.0252 (12)	0.0165 (11)
C5E	0.0559 (11)	0.0864 (15)	0.0708 (13)	0.0286 (11)	0.0005 (10)	−0.0058 (11)
C6E	0.0475 (10)	0.0652 (11)	0.0594 (11)	0.0129 (9)	0.0006 (8)	−0.0121 (9)
C0A	0.0817 (14)	0.0789 (14)	0.0427 (10)	0.0084 (11)	0.0131 (9)	0.0151 (9)
C1A	0.0488 (9)	0.0493 (9)	0.0372 (8)	0.0080 (7)	0.0101 (7)	0.0052 (7)
O1A	0.0455 (6)	0.0604 (7)	0.0344 (5)	0.0013 (5)	0.0050 (5)	0.0059 (5)
C2A	0.0483 (9)	0.0644 (11)	0.0406 (9)	0.0041 (8)	0.0111 (7)	0.0102 (8)
O2A	0.0480 (7)	0.0745 (9)	0.0454 (7)	0.0004 (6)	0.0022 (5)	0.0102 (6)
C3A	0.0514 (10)	0.0583 (10)	0.0417 (9)	0.0010 (8)	0.0100 (7)	0.0062 (7)
O3A	0.0604 (8)	0.0843 (10)	0.0394 (6)	0.0042 (7)	−0.0003 (6)	0.0168 (6)
C4A	0.0511 (9)	0.0525 (9)	0.0385 (8)	0.0031 (8)	0.0049 (7)	0.0041 (7)
C5A	0.0448 (9)	0.0660 (11)	0.0402 (8)	0.0023 (8)	−0.0001 (7)	0.0045 (8)
C6A	0.0461 (9)	0.0597 (10)	0.0420 (9)	0.0007 (8)	0.0069 (7)	0.0067 (7)
C7A	0.0531 (10)	0.0528 (9)	0.0377 (8)	0.0064 (8)	0.0012 (7)	0.0068 (7)
C8A	0.0483 (10)	0.0873 (14)	0.0501 (10)	−0.0044 (10)	−0.0049 (8)	0.0148 (10)
C9A	0.0487 (10)	0.0825 (14)	0.0519 (10)	−0.0060 (9)	0.0051 (8)	0.0168 (9)
C0B	0.207 (4)	0.120 (3)	0.0670 (17)	0.010 (3)	−0.045 (2)	0.0233 (17)
C1B	0.0479 (9)	0.0398 (8)	0.0381 (8)	0.0090 (7)	0.0038 (6)	−0.0010 (6)
O1B	0.0472 (6)	0.0516 (6)	0.0337 (5)	0.0011 (5)	0.0019 (4)	0.0033 (5)
C2B	0.0520 (9)	0.0471 (9)	0.0387 (8)	0.0045 (7)	0.0043 (7)	0.0005 (7)
O2B	0.0480 (7)	0.0566 (7)	0.0456 (6)	0.0011 (6)	0.0046 (5)	0.0009 (5)
C3B	0.0546 (10)	0.0488 (9)	0.0406 (8)	0.0073 (8)	0.0038 (7)	−0.0009 (7)
O3B	0.167 (2)	0.1158 (16)	0.0544 (10)	0.0039 (15)	−0.0111 (12)	0.0258 (10)
C4B	0.0635 (11)	0.0472 (9)	0.0388 (8)	0.0094 (8)	−0.0020 (7)	−0.0037 (7)
C5B	0.0700 (13)	0.0689 (12)	0.0456 (10)	0.0034 (10)	−0.0004 (9)	0.0030 (9)
C6B	0.0946 (17)	0.0814 (15)	0.0525 (12)	−0.0017 (13)	0.0122 (11)	0.0105 (10)
C7B	0.112 (2)	0.0635 (12)	0.0441 (10)	0.0060 (13)	−0.0051 (12)	0.0075 (9)
C8B	0.0881 (17)	0.0912 (17)	0.0564 (12)	0.0107 (14)	−0.0254 (12)	0.0035 (11)
C9B	0.0675 (13)	0.0846 (15)	0.0536 (11)	0.0051 (11)	−0.0071 (10)	0.0052 (10)
C1F	0.068 (5)	0.092 (5)	0.105 (7)	0.009 (4)	0.003 (4)	0.003 (4)
C2F	0.077 (5)	0.101 (8)	0.097 (7)	0.009 (5)	−0.007 (4)	0.010 (5)
C3F	0.070 (5)	0.070 (4)	0.135 (18)	−0.003 (4)	−0.009 (9)	0.013 (8)
C4F	0.087 (6)	0.101 (8)	0.149 (15)	0.013 (6)	0.030 (9)	−0.019 (8)
C5F	0.203 (13)	0.163 (11)	0.138 (9)	0.075 (10)	0.099 (9)	0.053 (8)
C6F	0.127 (9)	0.112 (6)	0.154 (9)	0.052 (6)	0.055 (7)	0.055 (7)
C1G	0.088 (5)	0.086 (5)	0.093 (6)	0.019 (4)	0.019 (5)	0.019 (5)
C2G	0.115 (11)	0.091 (8)	0.075 (8)	0.010 (6)	−0.026 (7)	0.012 (6)
C3G	0.099 (19)	0.15 (3)	0.081 (6)	−0.034 (15)	−0.003 (9)	−0.023 (12)
C4G	0.097 (11)	0.18 (4)	0.109 (18)	0.01 (2)	−0.012 (11)	−0.047 (17)
C5G	0.108 (11)	0.185 (14)	0.195 (18)	0.067 (10)	0.010 (10)	−0.058 (12)
C6G	0.156 (11)	0.111 (8)	0.146 (11)	0.063 (7)	−0.003 (8)	0.016 (7)

### Geometric parameters (Å, º)

**Table:**

Sb—C1E	2.1034 (16)	C5A—H5A	0.93
Sb—C1C	2.1042 (15)	C6A—C7A	1.380 (2)
Sb—O1A	2.1054 (11)	C6A—H6A	0.93
Sb—C1D	2.1177 (16)	C7A—C8A	1.381 (3)
Sb—O1B	2.1238 (11)	C8A—C9A	1.369 (3)
C1C—C6C	1.380 (2)	C8A—H8A	0.93
C1C—C2C	1.388 (2)	C9A—H9A	0.93
C2C—C3C	1.375 (3)	C0B—O3B	1.376 (4)
C2C—H2C	0.93	C0B—H0B1	0.96
C3C—C4C	1.371 (3)	C0B—H0B2	0.96
C3C—H3C	0.93	C0B—H0B3	0.96
C4C—C5C	1.370 (3)	C1B—O2B	1.222 (2)
C4C—H4C	0.93	C1B—O1B	1.3080 (19)
C5C—C6C	1.387 (3)	C1B—C2B	1.468 (2)
C5C—H5C	0.93	C2B—C3B	1.323 (2)
C6C—H6C	0.93	C2B—H2B	0.93
C1D—C2D	1.374 (2)	C3B—C4B	1.461 (2)
C1D—C6D	1.379 (3)	C3B—H3B	0.93
C2D—C3D	1.390 (3)	O3B—C7B	1.367 (3)
C2D—H2D	0.93	C4B—C9B	1.377 (3)
C3D—C4D	1.362 (3)	C4B—C5B	1.383 (3)
C3D—H3D	0.93	C5B—C6B	1.373 (3)
C4D—C5D	1.351 (3)	C5B—H5B	0.93
C4D—H4D	0.93	C6B—C7B	1.378 (4)
C5D—C6D	1.389 (3)	C6B—H6B	0.93
C5D—H5D	0.93	C7B—C8B	1.353 (4)
C6D—H6D	0.93	C8B—C9B	1.395 (3)
C1E—C2E	1.368 (3)	C8B—H8B	0.93
C1E—C6E	1.377 (2)	C9B—H9B	0.93
C2E—C3E	1.385 (3)	C1F—C2F	1.34 (2)
C2E—H2E	0.93	C1F—C6F	1.313 (19)
C3E—C4E	1.354 (4)	C1F—H1F	0.93
C3E—H3E	0.93	C2F—C3F	1.39 (2)
C4E—C5E	1.357 (3)	C2F—H2F	0.93
C4E—H4E	0.93	C3F—C4F	1.317 (15)
C5E—C6E	1.376 (3)	C3F—H3F	0.93
C5E—H5E	0.93	C4F—C5F	1.362 (14)
C6E—H6E	0.93	C4F—H4F	0.93
C0A—O3A	1.415 (2)	C5F—C6F	1.322 (14)
C0A—H0A1	0.96	C5F—H5F	0.93
C0A—H0A2	0.96	C6F—H6F	0.93
C0A—H0A3	0.96	C1G—C2G	1.393 (5)
C1A—O2A	1.214 (2)	C1G—C6G	1.389 (5)
C1A—O1A	1.311 (2)	C1G—H1G	0.93
C1A—C2A	1.473 (2)	C2G—C3G	1.397 (5)
C2A—C3A	1.314 (3)	C2G—H2G	0.93
C2A—H2A	0.93	C3G—C4G	1.397 (5)
C3A—C4A	1.463 (2)	C3G—H3G	0.93
C3A—H3A	0.93	C4G—C5G	1.398 (5)
O3A—C7A	1.357 (2)	C4G—H4G	0.93
C4A—C5A	1.380 (2)	C5G—C6G	1.395 (5)
C4A—C9A	1.391 (3)	C5G—H5G	0.93
C5A—C6A	1.383 (2)	C6G—H6G	0.93
			
C1E—Sb—C1C	140.19 (6)	C4A—C5A—H5A	118.8
C1E—Sb—O1A	92.88 (5)	C6A—C5A—H5A	118.8
C1C—Sb—O1A	89.43 (5)	C7A—C6A—C5A	119.24 (16)
C1E—Sb—C1D	107.48 (6)	C7A—C6A—H6A	120.4
C1C—Sb—C1D	112.33 (6)	C5A—C6A—H6A	120.4
O1A—Sb—C1D	86.57 (6)	O3A—C7A—C6A	124.72 (16)
C1E—Sb—O1B	91.28 (5)	O3A—C7A—C8A	115.87 (16)
C1C—Sb—O1B	91.12 (5)	C6A—C7A—C8A	119.41 (16)
O1A—Sb—O1B	172.81 (4)	C9A—C8A—C7A	120.50 (17)
C1D—Sb—O1B	86.60 (5)	C9A—C8A—H8A	119.8
C6C—C1C—C2C	120.57 (16)	C7A—C8A—H8A	119.8
C6C—C1C—Sb	116.60 (12)	C8A—C9A—C4A	121.47 (18)
C2C—C1C—Sb	122.83 (12)	C8A—C9A—H9A	119.3
C3C—C2C—C1C	119.15 (17)	C4A—C9A—H9A	119.3
C3C—C2C—H2C	120.4	O3B—C0B—H0B1	109.5
C1C—C2C—H2C	120.4	O3B—C0B—H0B2	109.5
C4C—C3C—C2C	120.32 (18)	H0B1—C0B—H0B2	109.5
C4C—C3C—H3C	119.8	O3B—C0B—H0B3	109.5
C2C—C3C—H3C	119.8	H0B1—C0B—H0B3	109.5
C5C—C4C—C3C	120.82 (18)	H0B2—C0B—H0B3	109.5
C5C—C4C—H4C	119.6	O2B—C1B—O1B	121.75 (14)
C3C—C4C—H4C	119.6	O2B—C1B—C2B	124.10 (15)
C4C—C5C—C6C	119.67 (19)	O1B—C1B—C2B	114.13 (14)
C4C—C5C—H5C	120.2	C1B—O1B—Sb	111.99 (10)
C6C—C5C—H5C	120.2	C3B—C2B—C1B	121.50 (16)
C1C—C6C—C5C	119.45 (18)	C3B—C2B—H2B	119.2
C1C—C6C—H6C	120.3	C1B—C2B—H2B	119.2
C5C—C6C—H6C	120.3	C2B—C3B—C4B	127.26 (17)
C2D—C1D—C6D	118.76 (16)	C2B—C3B—H3B	116.4
C2D—C1D—Sb	120.91 (13)	C4B—C3B—H3B	116.4
C6D—C1D—Sb	120.28 (13)	C7B—O3B—C0B	119.4 (3)
C1D—C2D—C3D	119.9 (2)	C9B—C4B—C5B	117.31 (18)
C1D—C2D—H2D	120	C9B—C4B—C3B	119.17 (19)
C3D—C2D—H2D	120	C5B—C4B—C3B	123.52 (17)
C4D—C3D—C2D	120.8 (2)	C6B—C5B—C4B	121.5 (2)
C4D—C3D—H3D	119.6	C6B—C5B—H5B	119.2
C2D—C3D—H3D	119.6	C4B—C5B—H5B	119.2
C5D—C4D—C3D	119.67 (19)	C5B—C6B—C7B	120.3 (2)
C5D—C4D—H4D	120.2	C5B—C6B—H6B	119.9
C3D—C4D—H4D	120.2	C7B—C6B—H6B	119.9
C4D—C5D—C6D	120.5 (2)	C8B—C7B—O3B	125.5 (2)
C4D—C5D—H5D	119.7	C8B—C7B—C6B	119.3 (2)
C6D—C5D—H5D	119.7	O3B—C7B—C6B	115.2 (3)
C1D—C6D—C5D	120.4 (2)	C7B—C8B—C9B	120.5 (2)
C1D—C6D—H6D	119.8	C7B—C8B—H8B	119.8
C5D—C6D—H6D	119.8	C9B—C8B—H8B	119.8
C2E—C1E—C6E	119.65 (17)	C4B—C9B—C8B	121.1 (2)
C2E—C1E—Sb	116.39 (13)	C4B—C9B—H9B	119.5
C6E—C1E—Sb	123.93 (13)	C8B—C9B—H9B	119.5
C1E—C2E—C3E	120.1 (2)	C2F—C1F—C6F	118 (2)
C1E—C2E—H2E	120	C2F—C1F—H1F	121
C3E—C2E—H2E	120	C6F—C1F—H1F	121
C4E—C3E—C2E	119.9 (2)	C3F—C2F—C1F	119.9 (17)
C4E—C3E—H3E	120.1	C3F—C2F—H2F	120.1
C2E—C3E—H3E	120.1	C1F—C2F—H2F	120.1
C3E—C4E—C5E	120.3 (2)	C2F—C3F—C4F	119.9 (14)
C3E—C4E—H4E	119.9	C2F—C3F—H3F	120
C5E—C4E—H4E	119.9	C4F—C3F—H3F	120
C4E—C5E—C6E	120.8 (2)	C5F—C4F—C3F	118.9 (10)
C4E—C5E—H5E	119.6	C5F—C4F—H4F	120.6
C6E—C5E—H5E	119.6	C3F—C4F—H4F	120.6
C5E—C6E—C1E	119.34 (19)	C6F—C5F—C4F	119.3 (9)
C5E—C6E—H6E	120.3	C6F—C5F—H5F	120.3
C1E—C6E—H6E	120.3	C4F—C5F—H5F	120.3
O3A—C0A—H0A1	109.5	C5F—C6F—C1F	123.2 (11)
O3A—C0A—H0A2	109.5	C5F—C6F—H6F	118.4
H0A1—C0A—H0A2	109.5	C1F—C6F—H6F	118.4
O3A—C0A—H0A3	109.5	C2G—C1G—C6G	119.3 (11)
H0A1—C0A—H0A3	109.5	C2G—C1G—H1G	120.4
H0A2—C0A—H0A3	109.5	C6G—C1G—H1G	120.4
O2A—C1A—O1A	122.33 (15)	C1G—C2G—C3G	121.7 (15)
O2A—C1A—C2A	121.57 (16)	C1G—C2G—H2G	119.2
O1A—C1A—C2A	116.09 (15)	C3G—C2G—H2G	119.2
C1A—O1A—Sb	113.18 (10)	C2G—C3G—C4G	118.2 (17)
C3A—C2A—C1A	124.28 (16)	C2G—C3G—H3G	120.9
C3A—C2A—H2A	117.9	C4G—C3G—H3G	120.9
C1A—C2A—H2A	117.9	C5G—C4G—C3G	120.7 (16)
C2A—C3A—C4A	127.75 (17)	C5G—C4G—H4G	119.7
C2A—C3A—H3A	116.1	C3G—C4G—H4G	119.7
C4A—C3A—H3A	116.1	C6G—C5G—C4G	119.8 (13)
C7A—O3A—C0A	117.89 (15)	C6G—C5G—H5G	120.1
C5A—C4A—C9A	117.04 (16)	C4G—C5G—H5G	120.1
C5A—C4A—C3A	123.78 (16)	C5G—C6G—C1G	120.2 (10)
C9A—C4A—C3A	119.16 (16)	C5G—C6G—H6G	119.9
C4A—C5A—C6A	122.33 (16)	C1G—C6G—H6G	119.9
			
C1E—Sb—C1C—C6C	−171.05 (11)	C1D—Sb—O1A—C1A	−175.72 (12)
O1A—Sb—C1C—C6C	95.13 (13)	O2A—C1A—C2A—C3A	−173.88 (19)
C1D—Sb—C1C—C6C	9.07 (14)	O1A—C1A—C2A—C3A	5.2 (3)
O1B—Sb—C1C—C6C	−77.71 (13)	C1A—C2A—C3A—C4A	178.64 (18)
C1E—Sb—C1C—C2C	9.60 (17)	C2A—C3A—C4A—C5A	3.2 (3)
O1A—Sb—C1C—C2C	−84.22 (13)	C2A—C3A—C4A—C9A	−178.6 (2)
C1D—Sb—C1C—C2C	−170.28 (12)	C9A—C4A—C5A—C6A	−1.3 (3)
O1B—Sb—C1C—C2C	102.95 (13)	C3A—C4A—C5A—C6A	176.94 (18)
C6C—C1C—C2C—C3C	−1.1 (2)	C4A—C5A—C6A—C7A	0.2 (3)
Sb—C1C—C2C—C3C	178.24 (12)	C0A—O3A—C7A—C6A	−3.3 (3)
C1C—C2C—C3C—C4C	1.3 (3)	C0A—O3A—C7A—C8A	177.63 (19)
C2C—C3C—C4C—C5C	−0.4 (3)	C5A—C6A—C7A—O3A	−178.01 (17)
C3C—C4C—C5C—C6C	−0.7 (3)	C5A—C6A—C7A—C8A	1.1 (3)
C2C—C1C—C6C—C5C	0.0 (3)	O3A—C7A—C8A—C9A	177.9 (2)
Sb—C1C—C6C—C5C	−179.39 (14)	C6A—C7A—C8A—C9A	−1.2 (3)
C4C—C5C—C6C—C1C	0.9 (3)	C7A—C8A—C9A—C4A	0.1 (4)
C1E—Sb—C1D—C2D	−112.33 (15)	C5A—C4A—C9A—C8A	1.1 (3)
C1C—Sb—C1D—C2D	67.58 (16)	C3A—C4A—C9A—C8A	−177.2 (2)
O1A—Sb—C1D—C2D	−20.39 (15)	O2B—C1B—O1B—Sb	2.65 (19)
O1B—Sb—C1D—C2D	157.39 (15)	C2B—C1B—O1B—Sb	−175.76 (10)
C1E—Sb—C1D—C6D	65.18 (16)	C1E—Sb—O1B—C1B	70.43 (11)
C1C—Sb—C1D—C6D	−114.90 (16)	C1C—Sb—O1B—C1B	−69.83 (11)
O1A—Sb—C1D—C6D	157.12 (16)	C1D—Sb—O1B—C1B	177.87 (11)
O1B—Sb—C1D—C6D	−25.10 (16)	O2B—C1B—C2B—C3B	−9.7 (3)
C6D—C1D—C2D—C3D	−0.5 (3)	O1B—C1B—C2B—C3B	168.66 (16)
Sb—C1D—C2D—C3D	177.10 (16)	C1B—C2B—C3B—C4B	−174.99 (16)
C1D—C2D—C3D—C4D	−0.7 (4)	C2B—C3B—C4B—C9B	170.5 (2)
C2D—C3D—C4D—C5D	1.4 (4)	C2B—C3B—C4B—C5B	−9.3 (3)
C3D—C4D—C5D—C6D	−0.8 (4)	C9B—C4B—C5B—C6B	−0.4 (3)
C2D—C1D—C6D—C5D	1.0 (3)	C3B—C4B—C5B—C6B	179.43 (19)
Sb—C1D—C6D—C5D	−176.57 (18)	C4B—C5B—C6B—C7B	−0.7 (4)
C4D—C5D—C6D—C1D	−0.4 (4)	C0B—O3B—C7B—C8B	0.2 (4)
C1C—Sb—C1E—C2E	−168.71 (14)	C0B—O3B—C7B—C6B	−178.7 (3)
O1A—Sb—C1E—C2E	−76.13 (16)	C5B—C6B—C7B—C8B	1.2 (4)
C1D—Sb—C1E—C2E	11.17 (17)	C5B—C6B—C7B—O3B	−179.8 (2)
O1B—Sb—C1E—C2E	98.01 (16)	O3B—C7B—C8B—C9B	−179.4 (2)
C1C—Sb—C1E—C6E	13.2 (2)	C6B—C7B—C8B—C9B	−0.6 (4)
O1A—Sb—C1E—C6E	105.73 (15)	C5B—C4B—C9B—C8B	1.1 (3)
C1D—Sb—C1E—C6E	−166.97 (15)	C3B—C4B—C9B—C8B	−178.8 (2)
O1B—Sb—C1E—C6E	−80.13 (15)	C7B—C8B—C9B—C4B	−0.6 (4)
C6E—C1E—C2E—C3E	−0.1 (3)	C6F—C1F—C2F—C3F	−4 (3)
Sb—C1E—C2E—C3E	−178.4 (2)	C1F—C2F—C3F—C4F	−5 (3)
C1E—C2E—C3E—C4E	0.9 (4)	C2F—C3F—C4F—C5F	8 (3)
C2E—C3E—C4E—C5E	−0.9 (4)	C3F—C4F—C5F—C6F	−3 (2)
C3E—C4E—C5E—C6E	0.3 (4)	C4F—C5F—C6F—C1F	−5 (2)
C4E—C5E—C6E—C1E	0.4 (3)	C2F—C1F—C6F—C5F	9 (3)
C2E—C1E—C6E—C5E	−0.5 (3)	C6G—C1G—C2G—C3G	2 (3)
Sb—C1E—C6E—C5E	177.59 (15)	C1G—C2G—C3G—C4G	−5 (4)
O2A—C1A—O1A—Sb	5.7 (2)	C2G—C3G—C4G—C5G	6 (4)
C2A—C1A—O1A—Sb	−173.38 (12)	C3G—C4G—C5G—C6G	−5 (3)
C1E—Sb—O1A—C1A	−68.37 (12)	C4G—C5G—C6G—C1G	3 (2)
C1C—Sb—O1A—C1A	71.87 (12)	C2G—C1G—C6G—C5G	−1 (2)
